# A systematic review: enhancing stroke recovery through complementary interventions—Clinical outcomes and neural activity insights

**DOI:** 10.3389/fnins.2024.1437130

**Published:** 2024-11-13

**Authors:** Umi Nabilah Ismail, Noorazrul Yahya, Wan Asyraf Wan Zaidi, Zhe Kang Law, Hanani Abdul Manan

**Affiliations:** ^1^Makmal Pemprosesan Imej Kefungsian (Functional Image Processing Laboratory), Department of Radiology, Faculty of Medicine, Universiti Kebangsaan Malaysia, Kuala Lumpur, Malaysia; ^2^Diagnostic Imaging & Radiotherapy Program, Centre of Diagnostic, Therapeutic and Investigative Sciences (CODTIS), Faculty of Health Sciences, Universiti Kebangsaan Malaysia, Kuala Lumpur, Malaysia; ^3^Neurology Unit, Department of Medicine, Faculty of Medicine, Universiti Kebangsaan Malaysia, Kuala Lumpur, Malaysia; ^4^Department of Radiology and Intervention, Hospital Pakar Kanak-Kanak (Children Specialist Hospital), Universiti Kebangsaan Malaysia, Kuala Lumpur, Malaysia

**Keywords:** stroke, complementary therapy, acupuncture, motor imagery, music, virtual reality, fMRI, neuroimaging

## Abstract

The growing interest in complementary interventions for stroke recovery necessitates the need for neural insights to aid in making evidence-based clinical decisions. This systematic review examined the brain activation effects of complementary therapies, including acupuncture (*n* = 5), motor imagery therapy (MIT) (*n* = 5), music (*n* = 3), and virtual reality (VR) interventions (*n* = 3), on clinical outcomes and neural activity in stroke patients. All therapies engaged motor and sensory networks, frontal regions, parietal regions, and temporal regions, suggesting their potential to improve motor control, attention, memory, and cognitive function. Acupuncture activated motor areas in both hemispheres, while MIT stimulated frontoparietal regions in both sides of the brain, supporting whole-body integration in recovery. In contrast, VR therapy exhibited ipsilesional lateralization, while music therapy showed left-lateralization. The review also found that increased interhemispheric connectivity between motor regions, along with intrahemispheric ipsilesional connectivity between motor, cognitive, and sensory areas, is key to achieving better clinical outcomes.

**Systematic Review Registration:**

http://www.crd.york.ac.uk/PROSPERO, identifier (ID: CRD42023455192).

## 1 Introduction

Stroke continues to be the world’s second-leading cause of death and the third-leading cause of death and disability (measured by disability-adjusted life-years lost, or DALYs) (Collaborators, 2021). Rehabilitation is a crucial component of stroke care, aimed at reducing disability and enhancing recovery (Maier et al., 2019). Standard rehabilitation approaches focus on physical and occupational therapy, but interest in complementary interventions to enhance stroke recovery is growing (Hart, 2010; Kadir et al., 2015).

Acupuncture, a traditional Chinese medicine practice, has been recognized by the World Health Organization for its potential role in stroke treatment. It works by stimulating specific points on the body, which activates the central nervous system (Chavez et al., 2017). While promising, acupuncture remains primarily an adjunctive therapy due to insufficient evidence from conflicting and methodologically weak studies (World Health Organization, 2002). Motor imagery has also been extensively studied for stroke rehabilitation. The concept behind motor imagery is that the regions activated during imagery tasks overlap significantly with those involved in actual movement execution. Thus, this therapy helps the affected areas of the brain engage in continuous training, promoting neuroplasticity in those regions (Guerra et al., 2017).

Virtual reality (VR), a novel approach to rehabilitation, immerses users in a synthetic three-dimensional environment. Compared to traditional methods, VR offers advantages such as high repetition, task specificity, objective feedback, and improved user engagement and motivation (Khokale et al., 2023). Repetition is essential for motor re-learning and neuroplasticity, which are key for improving stroke rehabilitation outcomes. Music therapy, which systematically uses musical elements as an intervention, has been shown to improve both neurological function and mood. Research suggests that musicians exhibit enhanced subcortical auditory and audiovisual processing. The high frequency of listening to or performing music can promote neural plasticity, regulate neural networks, and enhance motor function in stroke patients (Wan and Schlaug, 2010; Chatterjee et al., 2021).

Improved functional recovery post stroke is closely linked to the reorganization of the surviving central nervous system (Hodics et al., 2006; Grefkes and Fink, 2014; Hara, 2015). While the mechanisms behind various therapies have been studied, the key focus should be on how these therapies specifically influence brain activity, particularly in regions critical for stroke recovery. Understanding how these treatments activate important brain regions is essential for assessing their effectiveness in promoting neuroplasticity and functional recovery.

Functional magnetic resonance imaging (fMRI) is a non-invasive neuroimaging technique that that visualizes brain activity in response to different tasks or stimuli. It has been used to study the neural correlates of stroke rehabilitation interventions, providing insight into the changes in brain activation patterns associated with these interventions (Ward et al., 2003; Hodics et al., 2006). This systematic review examines complementary interventions—such as acupuncture, motor imagery therapy (MIT), music therapy, and VR—in terms of both their impact on functional recovery and their ability to activate brain regions critical for stroke recovery, as measured by fMRI.

## 2 Methods

### 2.1 Search strategy, study selection, and quality assessment

This systematic review was conducted following Preferred Reporting Items for Systematic Review and Meta-analysis (PRISMA) guidelines (Moher et al., 2009) and previous systematic reviews (Manan and Yahya, 2021; Hussein et al., 2022; Manan A. A. et al., 2022; Manan H. A. et al., 2022; Voon et al., 2023). One independent researcher (U.N.I) conducted a preliminary search on the PubMed/MEDLINE and Scopus electronic databases to identify relevant complementary interventions applied to stroke patients, with a specific focus on studies that reported results using fMRI. This preliminary search aimed to identify key interventions with available fMRI results. Interventions such as acupuncture, MIT, music, and VR were then identified.

Subsequently, a comprehensive article search was conducted, employing in-depth and detailed criteria to identify a comprehensive set of relevant studies whereby only articles published within the past 10 years were included. Search terms were as follow: (stroke) AND [acupuncture OR (“motor imagery”) OR (music) OR (“virtual reality”)] AND [(“functional magnetic resonance imaging”) OR (fMRI) OR (“functional MRI”)]. References from the relevant studies were also screened to identify additional primary studies that had not previously been identified.

**Figure 1:**
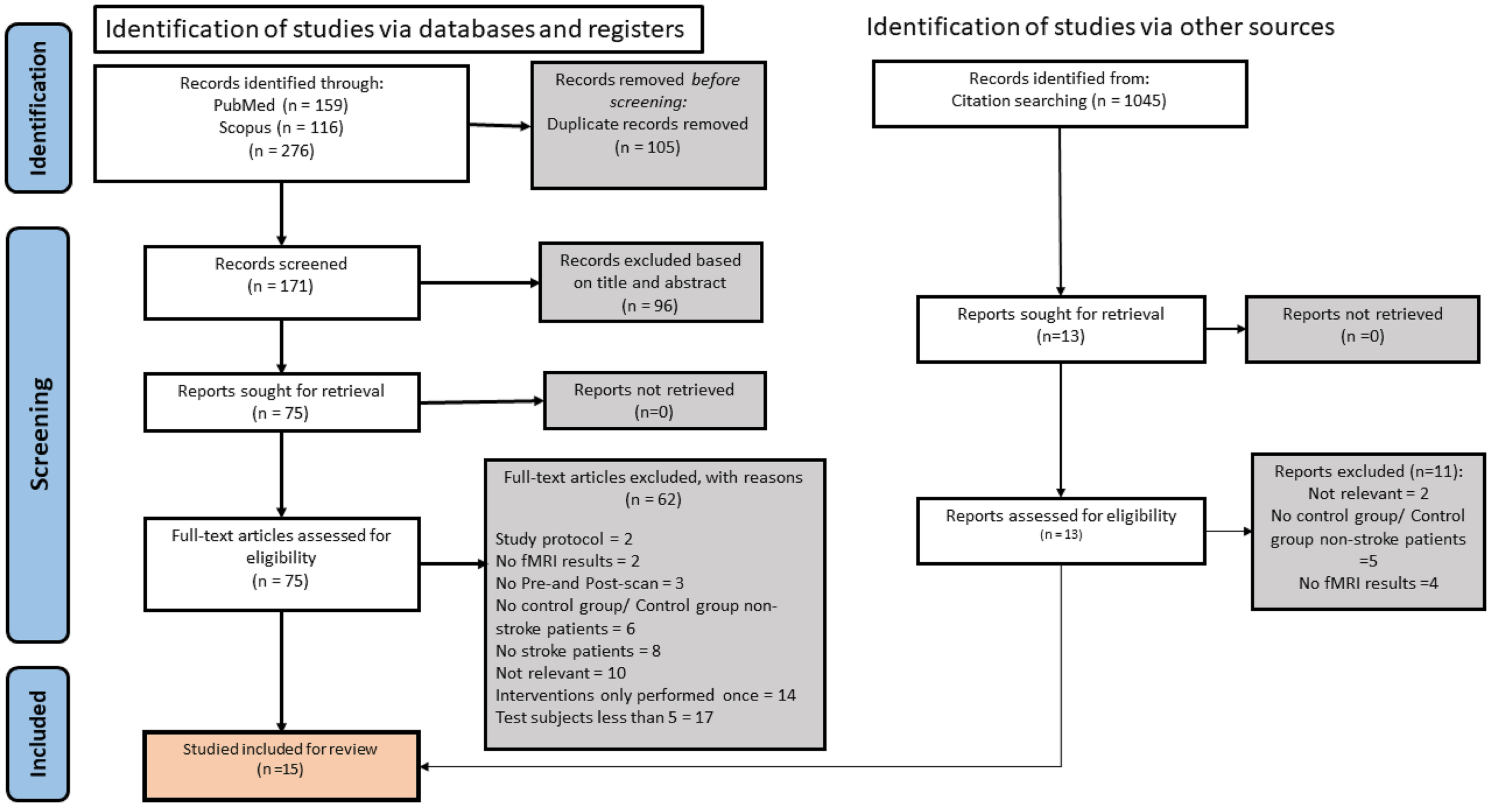
Flow diagram for study search and selection according to PRISMA guideline.

All screening and assessment procedures were carried out independently by two reviewers (U.N.I. and H.A.M) and consensus for eligibility was reached through discussion, using the PICOS strategy (Table 1) as guideline. The flow process of article selection for this review are detailed in Figure 1.

**Table 1:** PICOS characteristics.

Characteristics	Criteria
Population	Stroke patients (> 18 years old), regardless of lesion site, onset time or co-morbidities. The study must have more than 5 participants in the test group
Intervention	Adjunctive therapy such as music therapy, Motor imagery, Virtual reality, and Acupuncture Intervention must be more than one session.
Comparison	Compared to control group (stroke patients). Studies that had no control or baseline comparisons were excluded
Outcome	Studied were included if resting-fMRI or task-fMRI results are reported
Study type	Include all research study except all types of review, case study and case report.

### 2.2 Inclusion/exclusion criteria

A study must have fulfilled the following criteria: (1) interventions with at least more than one session; (2) subjects with stroke; to assess the effect of complementary interventions on the brain activities of stroke patients, both the control and experimental group must also be stroke patients who received conventional treatment; (3) studies with a sample size of at least 5 participants in the interventions group; (4) studies published in English; (5) the specific contribution of the complementary interventions must have been discernible, which excludes studies with combinations of complementary interventions were performed in the experimental group. All research studies except all types of reviews, case studies, and case reports using interventions such as acupuncture, MIT, music, and VR as complementary interventions were considered (Table 1).

Based on earlier systematic reviews, each intervention protocol’s inclusion and exclusion criteria are set (Furlan et al., 2005; Schuster et al., 2011; Baylan et al., 2016; Hao et al., 2022). This is to ensure the complementary intervention included in the articles are comparable across investigators. Specifically, for trials evaluating acupuncture, it must be specified by conventional acupuncture theory, and needles must be inserted in classical meridian points, additional points, or ah-shi points (painful sites) (Furlan et al., 2005). Since some studies have reported different brain activation patterns depending on the acupoint stimulation methods, studies that used acupuncture treatments like acupressure (Cho et al., 2010) or laser acupuncture (Quah-Smith et al., 2013) that did not involve dry needling were excluded. Intervention protocol with MIT required patients to practice mental imaging of specific movements or tasks. Studies were omitted if the imagery (1) is not related to movements, (2) based on a computer-animated technique, (3) carried out during hypnosis or psychotherapy, and (4) mental practice used as a mental rotation or diagnostic tool (Schuster et al., 2011). Trials evaluating music therapy included any music listening component, regardless of who provided the intervention, the intervention’s primary purpose, or the amount of intervention delivered (Baylan et al., 2016). For VR, studies focused on using immersive or non-immersive virtual reality in rehabilitation were included (Hao et al., 2022).

### 2.3 Risk of bias assessment

Since the included studies consist mostly of randomized controlled trials (RCT) studies, the Physiotherapy Evidence Database (PEDro) list was used to evaluate the risk of bias (Moseley et al., 2019). PEDRO consists of 10 items that are evaluated as present or absent. The presence of 10 of the items is tallied to calculate a summary score (called the total PEDro score, range 0 to 10; high scores denote low risk of bias).

### 2.4 Types of outcome measures

#### 2.4.1 Primary outcomes

The studies must include two fMRI measures in the intervention and control group before and after finishing the intervention treatment. The studies must report at least one of the resting-based fMRI, or task-based fMRI results. Only fMRI findings that compare the differences between control and experimental groups would be included in the study.

#### 2.4.2 Secondary outcomes

At least one of the pre-and post- interventions of neurological or psychological assessments was reported.

### 2.5 Data collection and analysis

Information about populations, interventions (method and dosage), populations, and primary and secondary outcomes were taken from papers. Conclusions were reached using a qualitative approach due to the heterogeneity and the insufficient number of comparable studies within the included subjects to conduct a quantitative meta-analysis.

## 3 Results

### 3.1 Search results

The initial search resulted in 276 potentially relevant articles being identified. One hundred five studies were eliminated as duplicates. The articles were then screened, whereby 96 articles were filtered out by title and abstract. From the remaining 75 articles, 61 articles were excluded based on the following: 17 articles had less than 5 subjects, 14 articles had a one-off session of interventions, 9 had objectives not relevant to the review, 8 did not have stroke patients as the subject, 6 had no control group or control group were non-stroke patients, 3 did not have both pre and post scanning, 2 did not use fMRI as the imaging technique, 2 were study protocol articles. Citation searching was performed on the relevant articles, and 2 articles were retrieved and included in the study. Overall, 16 studies met our inclusion criteria, 5 looked at the effects of acupuncture, 5 at the effects of motor imagery, 3 at the impact of music, and 3 at the effects of virtual reality on the brain’s activity in stroke patients using fMRI.

### 3.2 Quality assessment

The results of the quality assessment are shown in Table 2. All studies included were randomized controlled trial (RCT). In all studies, the patients were not blinded to the treatment allocation, in only one study the therapists were blinded while assessor were blinded in seven out of the 16 included studies. All articles showed a moderate and low risk of bias, making them suitable to be included in this review.

**Table 2:** Risk of bias assessment (Physiotherapy Evidence Database scale).

	Eligibility criteria	Random allocation	Concealed allocation	Baseline comparability	Subjects blinding	Therapist blinding	Assessor blinding	Adequate follow-up	Intention-to-treat analysis	Points estimates and measures of variability provided	Total PEDro score
Li et al. (2017)	**+**			**+**				**+**	**+**	**+**	**5/10**
Wu et al. (2017)	**+**	**+**	**+**	**+**				**+**	**+**	**+**	**7/10**
Liu et al. (2020), Liu et al. (2021)	**+**	**+**		**+**					**+**	**+**	**4/10**
Zhan et al. (2023)	**+**	**+**	**+**	**+**			**+**	**+**	**+**	**+**	**8/10**
Sun et al. (2013)	**+**	**+**		**+**			**+**	**+**	**+**	**+**	**7/10**
Liu et al. (2014)	**+**			**+**			**+**	**+**	**+**	**+**	**6/10**
Wang et al. (2019), Wang et al. (2020)	**+**	**+**		**+**			**+**	**+**	**+**	**+**	**7/10**
Wang et al. (2023)	**+**	**+**	**+**	**+**		**+**	**+**		**+**	**+**	**8/10**
Sihvonen et al. (2020), Sihvonen et al. (2021a), Sihvonen et al. (2021b)	**+**	**+**	**+**	**+**			**+**		**+**	**+**	**7/10**
Wang et al. (2017)	**+**	**+**		**+**				**+**	**+**	**+**	**6/10**
Mekbib et al. (2021)	**+**	**+**		**+**			**+**	**+**		**+**	**6/10**
Wu et al. (2022)	**+**			**+**				**+**	**+**	**+**	**5/10**

### 3.3 Studies characteristics

Patient characteristics summarized in Table 3 reveal that acupuncture trial had 97 ischemic stroke patients (mean age 50 to 70 years), MIT had 92 stroke patients (mean age 45 to 60 years), music therapy had 83 stroke patients (mean age 50 to 60 years), and VR therapy had 103 patients (mean age 50 to 60). There are five studies that focused exclusively on ischemic patients (all acupuncture and one VR study) while others recruited both types of stroke patients (Li et al., 2017; Wu et al., 2017; Alawieh et al., 2018; Liu et al., 2020; Liu et al., 2021; Wu et al., 2022; Zhan et al., 2023).

**Table 3:** Demographics of the included studies.

Study	Study design	Group	Sample size, n	Age (years)	Sex, n (M/F)	Lesion side, n (L/R)	Lesion location	Time of Onset	Type of stroke, n (H/I)	Other outcomes
Acupuncture										
Li et al. (2017)	CT	Acu	8	63.3 (12.3)	9/8	17/0	5 SC, 3 WM	56.1 (53.1) days	0/17	NDS
		Control	9				8 SC, 1 WM	41.6 (36.0) days		
		Healthy	14	62.2 (10.5)	8/6	–	–	–	–	–
Wu et al. (2017)	RCT	Acu	11	69.4(61.2–77.5)	7/4	na	21 SC	52.8(22.3–83.2) months	0/21	NDS, FMA, mBI
		Control	10	61.3(53.4–69.2)	5/5			52.2(18.0–86.4) months		
Liu et al. (2020), Liu et al. (2021)	RCT	Acu	6	59.7 (2.5)	4/2	13/0	13 SC	< 72 h	0/13	NIHSS, motor function scores
		Con	7	64.4 (7.4)	5/2					
Zhan et al. (2023)	RCT	Acu	24	57.7(8.9)	15/9	na	na	1.6(0.9) months	0/46	FMA, mBI, mRS
		Control	22	62.4(6.1)	15/7			2.1(1.2) months		
Motor imagery therapy(MIT)										
Sun et al. (2013)	RCT	MIT	9	56.7(12.7)	8/1	4/5	7 SC, 2 C	116.9(24.3) days	5/4	FM-UL
		Control	9	56.1(10.8)	9/0	5/4	8 SC, 1 C	132.1(27.3) days	4/5	
Liu et al. (2014)	RCT	MIT	10	45.8(4.4)	8/2	15/0	15 SC	1.6(09) months	na	FMA
		Control	5	45.0(4.3)	4/1			1.7(0.8) months		
Wang et al. (2019), Wang et al. (2020)	RCT	MIT	16	53.4(14.0)	16/0	9/7	30 SC	121.2(37.3) days	10/6	FM-UL, mBI
		Control	15	60.5(7.6)	14/1	9/6		164.7(74.8) days	7/8	
Wang et al. (2023)	RCT	MIT	15	56.7(10.7)	14/1	7/8	10 SC, 5 WM	118.5(35.5) days	7/8	FM-UL, mBI
		Control	13	61.8(4.5)	13/0	6/7	4 SC, 9 WM	143.2(46.6) days	6/7	
Music therapy (MT)										
Sihvonen et al. (2020)	RCT	Vocal MT	27	54.9(13.4)	15/12	15/12	na	6.1(2.6) days	na^**a**^	RBMT, VFT, BNT, CERAD, TT, CS, Stroop subtest, POMS
		Instrumental MT	23	56.7(10.3)	15/8	10/13		7.2(5.0) days		
		Audiobook MT	33	59.8(11.6)	16/17	16/17		7.3(4.1) days		
Sihvonen et al. (2021a), Sihvonen et al. (2021b)	RCT	Vocal MT	12	54.1(16.9)	5/7	6/6	na	na	2/10	
		Instrumental MT	15	53.6(10.3)	11/4	7/8			6/9	
		Audiobook MT	11	62.0(12.0)	7/4	7/4			4/7	
Virtual reality (VR)										
Wu et al. (2022)	CT	VR	22	52.0(12.4)	13/9	16/6	na	7.3(3.8) months	0/44	HAMD, mBI
		Control	22	50.7(10.0)	14/8	15/7		6.8(3.3) months		
Wang et al. (2017)	RCT	VR	13	53.4(7.7)	11/2	6/7	26 CS	7.2(2.0) weeks	na	WFMT
		Control	13	55.3(8.4)	11/2	8/5		7.9(2.1) weeks		
Mekbib et al. (2021)	RCT	VR	12	52.2(13.3)	9/3	na	na	1–3 months	9/3	FMA, mBI, MMSE
		Control	11	61.0(7.7)	8/3				8/3	
		Healthy	15	55.0(7.9)	–	–	–	–	–	

H, haemorrhage; I, Ischemic; SC, subcortical; C, cortical, WM, white matter; MCA, Middle Cerebellar Artery; NDS, Neurological Deficit Score; HAMD, Hamilton Depression; FMA, Fugl-Meyer Assessment; FM-UL, Fugl-Meyer Upper Limb; mRS, modified Rankin Scale; mBI, modified Barthel Index; NIHSS, National Institute Of Health Stroke Scale; RBMT, Rivermead Behavioural Memory Test; VFT, Verbal Fluency Task; TT, Token test; BNT, Boston Naming Test; CERAD, Consortium to Establish a Registry for Alzheimer’s Disease; CS, CogniSpeed© test battery; MBEA, Montreal Battery of Evaluation of Amusia; POMS, Profile of Mood States; WMF, Wolf Motor Function; MMSE, mini-mental state examination; na, not available; a refer to Sarkamo et al. (2014) and Sihvonen et al. (2021a).

Four studies (three acupuncture and one MIT studies) only included subjects with lesion at the left hemisphere (Liu et al., 2014; Li et al., 2017; Liu et al., 2020; Liu et al., 2021). Five studies two acupuncture, two MIT, and one VR. Three studies did not report lesion side and six studies did not report lesion location (Wu et al., 2017; Sihvonen et al., 2020; Mekbib et al., 2021; Sihvonen et al., 2021a; Sihvonen et al., 2021b; Zhan et al., 2023).

Five studies recruited subjects during acute phase (two acupuncture, and three music studies) (Liu et al., 2020; Sihvonen et al., 2020; Liu et al., 2021; Sihvonen et al., 2021a; Sihvonen et al., 2021b), nine studies during subacute (2 weeks to 6 months post-stroke) (two acupuncture, five MIT, two VR studies) (Sun et al., 2013; Liu et al., 2014; Li et al., 2017; Wang et al., 2017; Wang et al., 2019; Wang et al., 2020; Mekbib et al., 2021; Wang et al., 2023; Zhan et al., 2023), and two studies during chronic stage (more than 6 months post-stroke) (one acupuncture and one VR studies) (Wu et al., 2017; Wu et al., 2022).

**Figure 2:**
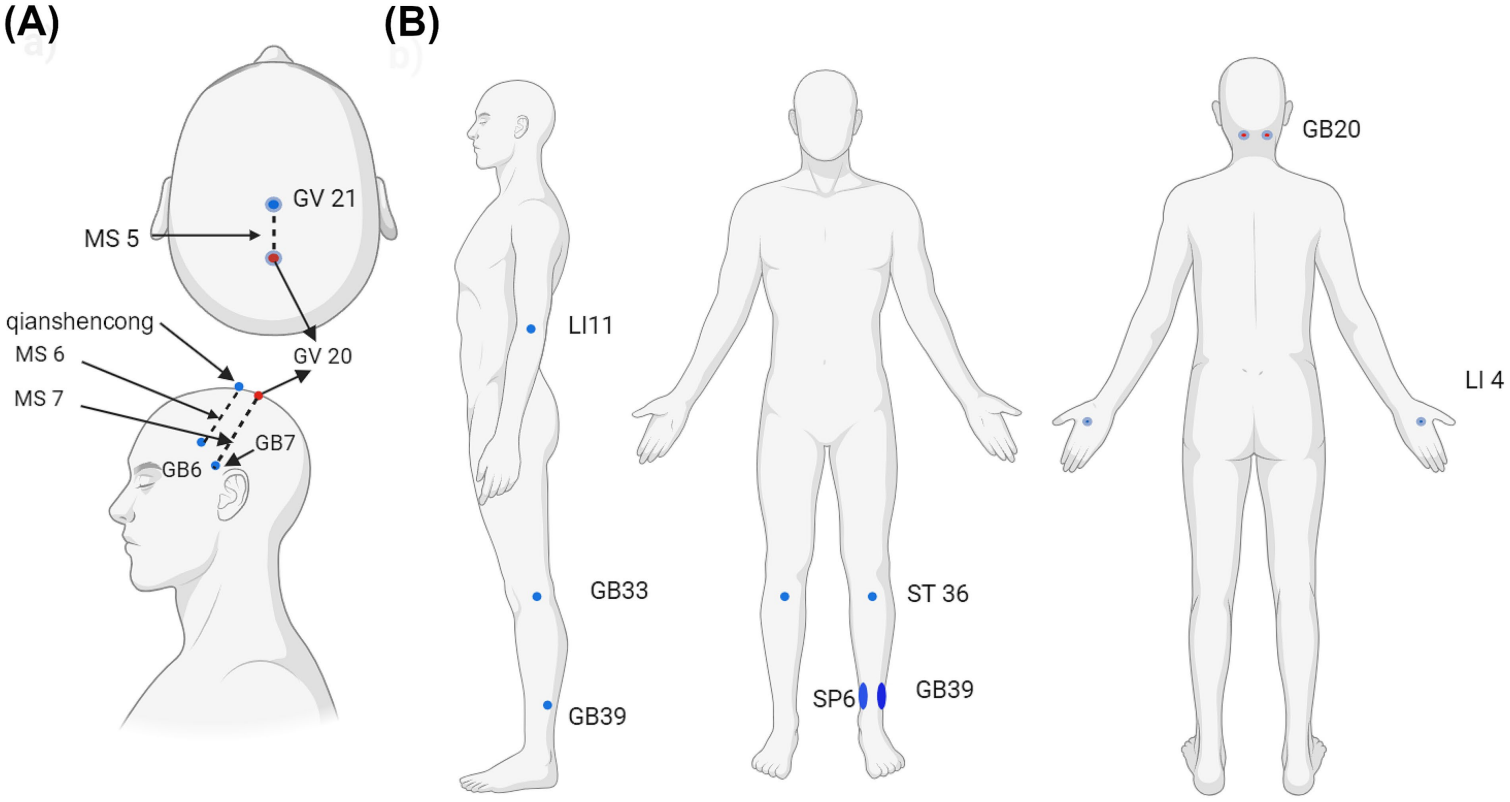
Acupoints used in the articles selected. (a) International Standardized Scalp Acupoints (ISSA) included MS5, MS6, MS7, Qianshencong [1 cun anterior to GV20 (Baihui) obliquely to GB6 (Xuanli)]. (b) Acupoints used for motor dysfunction included Baihui (GV 20), Fengchi (GB20, bilateral), Xuanzhong (GB 39), bilateral, Quchi (LI- 1 bilateral), Hegu (LI 4, bilateral), Zusanli (ST 36, bilateral), and Sanyinjiao (SP 6, bilateral). Created with BioRender.com.

On the methods used, two studies focused on acupoints related to treating motor dysfunction (Li et al., 2017; Wu et al., 2017) while the rest utilized the International Standard Scalp Acupuncture system (Liu et al., 2020; Liu et al., 2021; Zhan et al., 2023) (Figure 2). Notably, multiple publications involving same patients dataset, but with different analysis method were identified. All acupuncture subjects received conventional or modern medical treatment, including antiplatelet aggregation drugs, lipid-lowering and stabilizing agents, and blood pressure medication, as required by the patients. Details of the intervention were documented in Table 4.

**Table 4:** Detail of intervention for each study.

	Intervention	Comparison	Dosage
		Control group	
Acupuncture			
Li et al. (2017)	Acupuncture + conventional western medicine Acupoints: Baihui (GV 20), Fengchi (GB20, bilateral), Xuanzhong (GB 39), bilateral, Quchi (LI- 1 bilateral), Hegu (LI 4, bilateral), Zusanli (ST 36, bilateral), and Sanyinjiao (SP 6, bilateral)	Conventional western medicine only	Acu: 4 course, 5d/course, 2 h/d
Wu et al. (2017)	Acupuncture + standard treatment Acupoints: Baihui (GV 20), Fengchi (GB 20), Quchi (LI 11), Hegu (LI 4), Zusanli (ST 36), Yanglinquan (GB 33), Xuanzhong (GB 39), Sanyinjiao SP6	Standard treatment only	Acu: 4 course, 5 d/course, 30 min/sess
Liu et al. (2020), Liu et al. (2021)	Scalp acupuncture + Modern medicine Acupoints: MS5, left MS6, MS7	Modern medicine only	Acu: 1 course, 6d, 2 sess/d, 30 min/sess
Zhan et al. (2023)	Scalp acupuncture + standard treatment Acupoints: Ipsilesional MS6 area, Qianshencong (1 cun anterior to GV20 (Baihui) obliquely to GB6 (Xuanli))	Standard treatment only	Acu: 2 course, 8 w, 5 sess/w
Motor imagery			
Sun et al. (2013), Wang et al. (2019), Wang et al. (2020)	CRT + MI Imagine: In a warm relaxing place Perform simple flexion/ extension of the affected upper limb. Perform familiar activities of daily living. Refocus onto the room.	CRT	CRT: 4w, 5d/w, 3 h/d MI: CRT + 30 min
Wang et al. (2023)		patient-physician consultation/ health education	CRT: 4w, 5d/w, 3 h/d
			MI: CRT + 30 min
Liu et al. (2014)	Mental practice + MI + Physical Practice (PP) Imagine: Flexion/extension of thumb Abduction/adduction of all digits Making a fist/spreading the hand Moving extended fingers backwards and forwards, Moving the hand between the ulnar and radial deviation	Only PP	PP: 4 w, 5 d/w, 45 min/d MI: PP + 45 min
Music therapy			
Sihvonen et al. (2020), Sihvonen et al. (2021a), Sihvonen et al. (2021b)	Vocal MG: vocal music with sung lyric Instrumental MG: instrumental music Audiobook MG: narrated audiobook (without music)		2 m, 1 h/d.
Virtual reality			
Wang et al. (2017)	Physical and occupation therapy		
	Immersive-VR	2D VR	4w, 5d/w, 20 min/d
Wu et al. (2022)	Conventional physiotherapy		4w, 5d/w, 45 min/d
	Leap motion-based VR	Conventional occupational	4w, 5d/w, 45 min/d
Mekbib et al. (2021)	HTC Vive and Leap Motion VR + Occupational therapy (OT)	OT	OT: 2w, 4d/w, 1 h/d VR: + 1 h

For the MIT method used, four of the five investigations employed identical motor imagery task (Table 4). The control group of these four studies was only administered conventional rehabilitation therapy (CRT). The CRT includes acupuncture, electrical stimulation, physical therapy, and occupational therapy. Liu et al. (2014) subjected the control group to physical practise training and Neurodevelopmental Treatment-Bobath (NDT-Bobath) method.

All three studies included in this article employed the same method for music therapy (Table 4). Two articles published by Sihvonen et al.(Sihvonen et al., 2021a; Sihvonen et al., 2021b) reported a subset of patient data from the previous study (Sihvonen et al., 2020). Three-parallel arm RCT was employed whereby the patients were grouped into vocal music group (VMG), instrumental group (IMG), and audiobook group (ABG).

. Two articles used leap motion-based VR; the control group received either conventional occupational (Wang et al., 2017) or occupational therapy (Mekbib et al., 2021). Wu et al. (2022) used immersive VR, and the control group received 2D VR (Table 4).

### 3.4 Neurophysiological assessment

Better clinical outcomes in the acupuncture group than control were reported after acupuncture. Specifically, a greater decrease in neurological deficit scores (NDS) (Li et al., 2017; Wu et al., 2017) and national institute of health stroke scale (NIHSS) (Liu et al., 2020; Liu et al., 2021) and a greater increase in Fugl-Meyer assessment (FMA) and modified Barthel Index (mBI) (Wu et al., 2017; Zhan et al., 2023) were found in intervention group. One study reported no significant differences in FMA improvement between acupuncture and control group (Liu et al., 2020; Liu et al., 2021).

Only Sun et al. (2013) and Wang et al. (2020) reported no significant differences for FMA and MBI improvement between the MIT and control group, respectively. Other MIT studies showed significantly greater changes of FMA in MIT group.

Sihvonen et al. (2020) demonstrated that vocal music group had greater language skills and verbal memory improvement than audiobook and instrumental music group.

Two articles showed greater motor improvement in the VR than in the control group [wolf motor function test (Wang et al., 2017) and FMA (Mekbib et al., 2021)]. Study comparing immersive VR and 2D VR showed that though there was no difference in motor improvement, the immersive VR group demonstrated a greater decrease in Hamilton depression scale.

### 3.5 Brain activation and connectivity patterns

**Figure 3:**
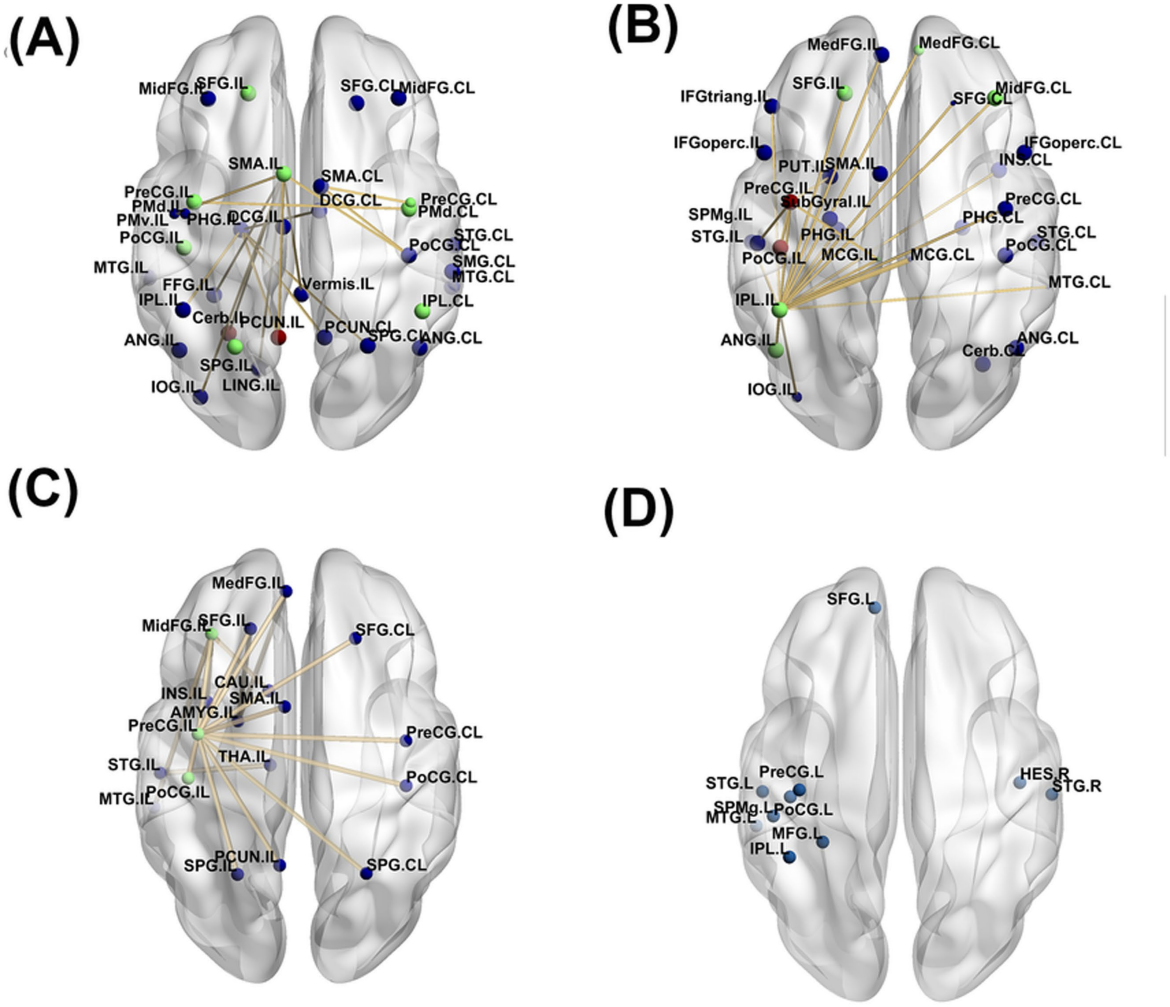
Significant brain alterations reported in the included studies for the following complementary therapies: (a) acupuncture, (b) melodic intonation therapy (MIT), (c) virtual reality (VR), and (d) music therapy. The lines represent functional connectivity changes, and the color of the dots indicates the number of times a region was reported as activated post-treatment. Blue dots represent regions identified in 1 study, green dots in 2 studies, and red dots in 3 or more studies.

Acupuncture studies included in this review demonstrated the ability of the treatment to engage both of the ipsilesional and contralesional hemisphere (Figure 3a). Motor regions such as precentral gyrus (PreCG), postcentral gyrus (PoCG), supplementary motor area (SMA), and dorsal and ventral premotor area (PMd and PMv) in both hemisphere were engaged with each other post acupuncture, reflecting a focus on motor control (Li et al., 2017; Wu et al., 2017; Zhan et al., 2023). Activations of the frontal regions included superior frontal gyrus (SFG) and middle frontal gyrus (MidFG) (Wu et al., 2017; Liu et al., 2020; Liu et al., 2021; Zhan et al., 2023). Activated and increased connections to the parietal regions such as inferior parietal lobule (IPL), supramarginal gyrus (SpMG), angular gyrus (ANG), and superior parietal gyrus (SPG), indicating acupuncture helps in attention and sensory processing (Wu et al., 2017; Liu et al., 2020; Liu et al., 2021; Zhan et al., 2023). Activation of superior temporal gyrus (STG) and middle temporal gyrus (MTG) in the temporal regions was also reported in two acupuncture studies (Wu et al., 2017; Liu et al., 2021). Additionally, the increased connection with parahippocampal gyrus (PHG), lingual gyrus (LING), inferior occipital gyrus (IOG), and fusiform gyrus (FFG) reflected acupuncture ability to stimulate visual and spatial processing (Liu et al., 2020). The increased connection with precuneus (PCUN), middle cingulate gyrus (MCG), cerebellum (Cerb) and vermis further indicate acupuncture promotes complex motor coordination, emotional processing, sensory integration and visuospatial awareness (Liu et al., 2020; Zhan et al., 2023).

The ability of MIT to activate and promote connections between the two hemisphere were almost similar to acupuncture (Figure 3b). Motor regions reported to activate post MIT training were PreCG, PoCG, and SMA (Sun et al., 2013; Liu et al., 2014; Wang et al., 2019; Wang et al., 2020; Wang et al., 2023). Specifically, increased activity with ipsilesional PreCG or decreased activity with contralesional PreCG was reported in four studies (Sun et al., 2013; Liu et al., 2014; Wang et al., 2019; Wang et al., 2023). Sun et al. (2013) showed that MIT reorganization patterns could occur in two ways; (1) increased activation of the ipsilesional PreCg and PoCG or (2) decreased activation of the ipsilesional PreCG and PoCG accompanied by an increased of PreCG and ProCG laterality index. MIT promoted higher connections to the frontal regions including SFG, medial frontal gyrus (MedFG), MidFG, inferior frontal gyrus (IFG), encompassing both the opercular (IFGoperc) and triangular (IFGtriang) parts (Wang et al., 2020).The parietal regions engaged were IPL, SpMG and ANG (Liu et al., 2014; Wang et al., 2020; Wang et al., 2023). The temporal regions reported were similar to acupuncture which were STG and MTG (Wang et al., 2020). Increased connection with PHG related with memory encoding and spatial processing (Wang et al., 2020). Additionally, increased connection with regions such as insula, MCG, putamen (PUT), and SubGyral indicated MIT stimulated emotional regulation and interoceptive process (Wang et al., 2019; Wang et al., 2020; Cheng et al., 2021; Wang et al., 2023). Similar to acupuncture, Cerb was also activated due to MIT activity (Liu et al., 2014).

The regions activated and connected due to VR showed strong ipsilesional-lateralization compared to acupuncture and MIT (Figure 3c). Motor regions reported were similar to MIT which were PreCG, PoCG, and SMA. Frontal regions reported include SFG, MidFG, and MedFG. The only parietal region connected were SPG. Temporal regions such as STG and MTG were only activated in the ipsilesional hemisphere. Other regions such as PCUN, amygdala, insula and caudate indicated that VR therapy may have a strong emotional, motivational or motor coordination component.

Music therapy similar with VR, had a very strong lateralization to one side of the hemisphere (Figure 3d) (Sihvonen et al., 2020). PreCG and PoCG were activated in the left hemisphere post music therapy. The study reported strong engagement between default mode network (DNM) regions with bilateral temporal regions such as STG, MTG, and Heschl’s gyrus while language network regions had strong connections with parietal regions such as IPL and SpMG. Vocal music were shown to cause greater engagement to the frontal regions like SFG and MidFG.

**Figure 4:**
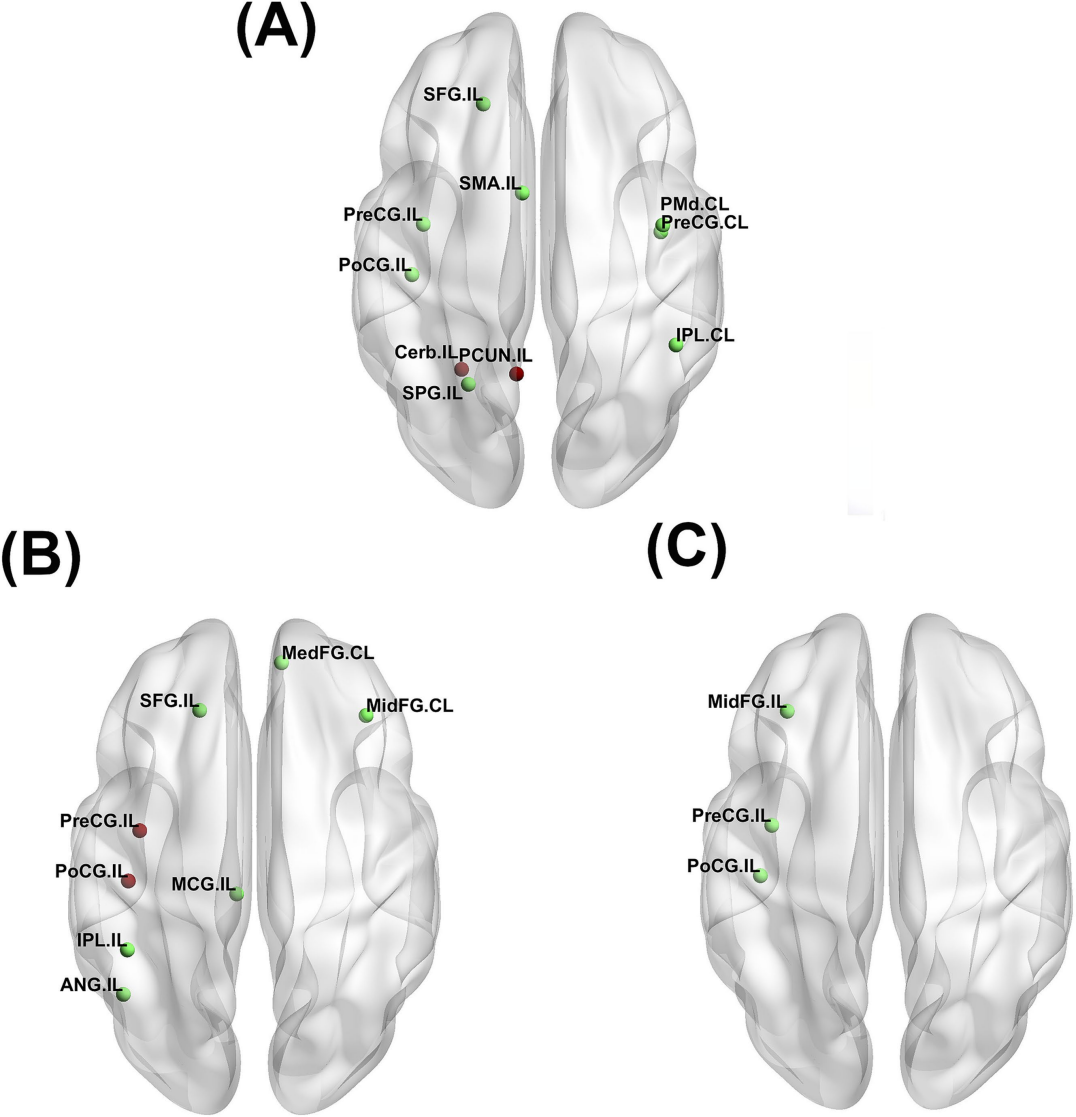
Significant brain alterations consistently reported in more than one study for the following complementary therapies: (a) acupuncture, (b) melodic intonation therapy (MIT), (c) virtual reality (VR). Green dots represent regions identified in 2 studies, while red dots represent regions identified in 3 or more studies. Only regions reported in multiple studies from different dataset are highlighted to emphasize consistent findings.

To provide a detailed evaluation of the regions activated post-treatment, Figure 4 highlights only those regions reported in more than one study. Music therapy was excluded in this analysis as the results obtained are from same dataset or subset of the data. The Cerb and PCUN were the most frequently activated regions, appearing in three studies related to acupuncture (Figure 4a). Motor regions in both hemispheres were reported as engaged in two studies. Additional activated areas include the ipsilesional SPG and SFG, as well as contralesional IPL. Regarding MIT, three studies reported engagement of the ipsilesional PreCG and PoCG. Other regions frequently reported were frontal regions (ipsilesional SFG and contralesional MedFG and MidFG), parietal regions (ipsilesional IPL and ANG), and ipsilesional MCG (Figure 4b). In studies involving virtual reality (VR), common activations were observed in the ipsilesional PreCG, PoCG, and MidFG (Figure 4c).

### 3.6 Correlations between brain activity and clinical outcomes

Significant correlation were found between changes in NDS with baseline FC of bilateral preCG (Li et al., 2017), and between changes in FMA with baseline Fractional amplitude of low-frequency fluctuations (fALFF) of contralesion PreCG and PoCG (Zhan et al., 2023).

Sun et al. (2013) and Liu et al. (2014) showed that increased activation of ipsilesional S1 and SMC and decreased activation of contralesional M1 correlated with improvement of FMA scores. Decreased FC of ipsilesional IPL with ipsilesional PreCG (Wang et al., 2023), bilateral MCG, and contralesional MedFG (Wang et al., 2020) were correlated with FMA scores improvement. Increased FC of ipsilesional PreCG with PUT (Wang et al., 2023) and ipsilesional IPL with bilateral PHG (Wang et al., 2020) were also associated with increased FMA scores. Notably, one study showed that an increased clustering coefficient was positively correlated with FMA scores (Wang et al., 2019).

Increased FC in left temporal regions with DMN and increased white matter volume in contralesional medial parieto-occipital areas of aphasic patients were correlated with language and verbal memory (Sihvonen et al., 2020). Increased FC in left IPL and PoCG with language network was associated with improvement in verbal memory (Sihvonen et al., 2021a). The increased activity in the left frontal cluster (SFG, MidFG and PreCG) of the was correlated with the increased fractional anisotropy in the left frontal aslant tract. No significant correlations were observed between brain activity and clinical outcomes in VR studies.

## 4 Discussion

The present systematic review aimed to investigate the effects of acupuncture, music therapy, virtual reality, and motor imagery on brain activation and connectivity patterns in stroke patients, as assessed through fMRI. The findings revealed that patients undergoing these therapies generally experienced improved clinical outcomes. All therapies engaged motor and sensory networks (PreCG, PoCG, SMA), frontal regions (MidFG, SFG), parietal regions (IPL), and temporal regions (STG, MTG), indicating their potential to enhance motor control, attention, memory, and cognitive functions. Acupuncture activated motor areas in both hemispheres, while MIT stimulated frontal regions in both sides of the brain, supporting whole-body integration in recovery. In contrast, VR therapy exhibited ipsilesional lateralization, while music therapy showed left-lateralization. The review also found that increased interhemispheric connectivity between motor regions, along with intrahemispheric ipsilesional connectivity between motor, cognitive, and sensory areas, is key to achieving better clinical outcomes (see Table 5)

**Table 5:** Summary of the effects of the interventions on stroke patients.

	Study	Principle findings			
		Regioms	fMRI	Clinical outcome	Correlation
Acupuncture					
1.	Li et al. (2017)	iSMA-cPMd, iSMA-cPoCG, cSMA-cPMd, cSMA-cPoCG	FC restored to normal (decrease) in both groups.	Greater NDS improvement in ACU than CON	Baseline fc M1-M1 correlate with ∆NDS (*r* = 0.579)
		iPreCG-cPreCG	FC restored to normal (increase) in ACU but lower than normal (decrease) in CON		
		iPMd-iPMv	FC higher than normal in ACU but lower than normal in CON		
2.	Wu et al. (2017)	PreCG	ReHo increased in both groups	NDS decreased while FMA and MBI increased Greater NDS and MBI improvement in ACU than CON	
		PoCG, SPL	ReHo increased in CON only		
		MidFG, biSpMG, MTG (BA21), Cerb	ReHo increased in ACU only		
3.	Liu et al. (2020)	iSMA- iPCUN, iLING, iFFG, iCerb, iDeclive, iVermis, iSPL, iIOG iPHG-iIPL, iPCUN, iIPG, iPCL, cMidFG, cSPG, cDCG, cPCUN	Increased FC in ACU only Result in CON not reported	Greater NIHSS improvement in ACU than in CON No significant difference between ACU and CON in motor function scores Improvement	
4.	Liu et al. (2021)	biSFG	Greater VHMC enhancement in ACU than CON	Greater NIHSS improvement in ACU than in CON No significant difference between ACU and CON in FM-UL improvement	
		cSTG	Greater ALFF enhancement in ACU than CON		
		cMTG, cIPL	Greater ReHo enhancement in ACU than CON		
5.	Zhan et al. (2023)	cANG, cIPL, PCUN, cPreCG, cPoCG, iSFG, iCerb	Greater fALFF in ACU than CON	Greater FMA, MBI and mRS improvement in ACU than in CON	Baseline fALFF value of ipsilateral PreCG correlate with ∆FMA
Motor imagery (MIT)					
1.	Wang et al. (2019)	iM1-iPreCG, iPoCG, iMCG, iSpMG	Intrahemispheric FC increased in MIT but decrease in CON	Greater FM-UL improvement in MIT than in CON	∆Clustering coefficient in MIT correlate with ∆FM-UL
2.	Wang et al. (2020)	iIPL-biPHG, biMedFG, iMidCG, iSTG, iSFG, iMTG, iANG, cPreCG, cMidFG, cMTG	FC increased in MIT but decreased in CON	Greater FM-UL improvement in MIT than in CON. No difference in MBI between groups	∆FC iIPL-biPHG positively correlated with ∆FM-UL
		iIPL-biMCG, cSFG, cMeFG, cINS, iIOG	FC decreased in MIT but increased in CON		∆FC iIPL – biMCG, cMeFG negatively correlated with ∆FM-UL
3.	Sun et al. (2013)	iSMC (PreCG and PoCG)	Activation increased in 66.7% of MIT and 55.6% of CON groups. Activation decreased but LI-SMC increased dramatically in 33.7% of MIT and 11.1% of CON group	FM-UL improved. No significant difference between groups.	Increased ∆iSMC correlate with ∆FM-UL
4	Liu et al. (2014)		ME: Greater activation increased in ipsiS1 in MIT than in CON MI: Activation in ipsiS1 increased while contraM1 decreased in MIT only MI: contraCrb and corpus collousm activated	Greater increased of ARAT in MIT than in CON ME: ∆ipsiS1 ∝ FMa in MIT only MI: ∆ipsiS1 and ∆contra M1 ∝ FMa	
5	Wang et al. (2023)	cS1 and iM1	Activation decrease significantly in MIT than in CON Negatively correlated with ∆FM-UL improvement	Greater FM-UL improvement in MIT than in CON Only MIT showed significant improvement of mBI.	
		iM1 - iIPL	Decrease significantly in MIT Negatively correlated with ∆FM-UL improvement		
		iM1 - iPUT	Increase significantly in MIT Positively correlated with ∆FM-UL improvement		
Music					
**1.**	Sihvonen et al. (2020)	STG, MTG - DMN	Increased FC greater in VMG than in ABGand IMG	Language skills and verbal memory improved more in VMG than in ABG, IMG.	∆FC ∝ improvement in language and verbal memory in VMG.
**2.**	Sihvonen et al. (2021a)	IPL, PoCG - LN	Increased FC greater in VMG than in ABG		
		IPL, PoCG, SpMG -LN	Increased FC greater in IMG than in ABG		
**3.**	Sihvonen et al. (2021b)		Significant task-fMRI predictors for language skills improvement Cluster 1: left SPMg, IPL and PoCG Cluster 2: bilateral SMA and CG		
Virtual reality (VR)					
**1.**	Mekbib et al. (2021)		Pre-VR: M1 seed only connected intra-hemispherically (S1, SPG, SFG, STG, SMA and contraCerebellum) Post-VR, FC of M1 with contralesional brain regions were re-established (M1,S1,SPG,SFG)	∆FM-UL greater in VR than in CON.	
**2.**	Wu et al. (2022)	dLPFC ➔ Amyg, INS, MTG, CAU mPFC ➔ INS pSTS ➔ THA	Increased FC in immersive VR but decrease in CON Increased directional regulation to dLPFC and mPFC	HAMD decrease more in immersive VR	
**3.**	Wang et al. (2017)		Greater increased of iSMC and LI-iSMC in VR than in CON No changes in SMA and Crb activation	Greater WFMT scores improvement and WFMT time shorter in VR than in CON.	

i, ipsilesional; c, contralesional; bi, bilateral; NDS, Neurological Deficit Score; HAMD, Hamilton Depression; FMA, Fugl-Meyer Assessment; FM-UL, Fugl-Meyer Upper Limb; mRS, modified Rankin Scale; mBI, modified Barthel Index; NIHSS, National Institute Of Health Stroke Scale; RBMT, Rivermead Behavioural Memory Test; VFT, Verbal Fluency Task; TT, Token test; BNT, Boston Naming Test; CERAD, Consortium to Establish a Registry for Alzheimer’s Disease; CS, CogniSpeed© test battery; MBEA, Montreal Battery of Evaluation of Amusia; POMS, Profile of Mood States; WMF, Wolf Motor Function; MMSE, mini-mental state examination; na, not available SMA, supplementary motor area; PMd, dorsal premotor area; PMv, ventral premotor area; PoCG, postcentral gyrus; PreCG, precentral gyrus; M1, primary motor cortex (PreCG); S1, somatosensory motor cortex (PoCG); SPL/SPG, superior parietal lobe/gyrus; IPL/IPG, inferior parietal lobe/gyrus; SPMg, supraparietal marginal gyrus; SFG, superior frontal gyrus; MidFG/MFG, middle frontal gyrus; MedFG, medial frontal gyrus; dLPFC, dorsolateral prefrontal cortex; mPFC, medial prefrontal cortex; STG, superior temporal gyrus; MTG, middle temporal gyrus; pSTS, posterior superior temporal sulcus; Cerb, cerebellum; PCUN, precuneus; LING, lingual gyrus; FFG, fusiform gyrus; IOG, inferior occipital gyrus; PHG, parahippocampal gyrus; DCG, dorsal cingulate cortex; MCG, middle cingulate cortex; PCL, paracental lobule; ANG, angular gyrus; INS, insula; PUT, putamen; AMYG, amygdala; CAU, caudate; THA, thalamus; DMN, default mode network; LN, language network.

### 4.1 Patients’ demographics and clinical outcomes

Optimizing the brain plasticity time window is crucial for effective stroke recovery (Dobkin, 2004; Saver, 2006; Cramer, 2008; Coleman et al., 2017). Adjunctive treatments are designed to enhance neuroplasticity in patients. However, several factors—such as age, stroke type, lesion side, and lesion location—have been recognized to influence recovery (Shelton and Reding, 2001; Gialanella et al., 2013; Yoo et al., 2020; Deb-Chatterji et al., 2022). Most studies included in this review did not establish upper age limits or specify lesion side, stroke type, or lesion location in their inclusion/exclusion criteria. The randomization process used during patient allocation helped ensure that treatment and control groups had comparable baseline characteristics, suggesting that the interventions’ effects on clinical outcomes and neural activity in subcortical stroke patients were not significantly influenced by these factors. Thus, organizing this review based on clinical variables, such as stroke type or lesion classification, might be less informative for readers.

The effectiveness of a treatment is influenced by the quality of its delivery and implementation. To achieve optimal results, standardizing treatment protocols is essential, ensuring consistency, repeatability, and comparability across studies. While standard clinical treatments in hospitals are often governed by established guidelines, adjunctive therapies frequently lack such standards. Nevertheless, as detailed in the review, despite variations in application methods, these therapies adhere to their core mechanisms. For instance, music therapy (Baylan et al., 2016; Huang et al., 2021; Zhang et al., 2021) and MIT (Batula et al., 2017; Guerra et al., 2017) show considerable differences in how they are delivered to stroke patients, leading to variations in treatment outcomes and brain activation patterns.

### 4.2 Lateralization of the brain

Stroke is known to reduced neural activity in the ipsilesional hemisphere and hypercitability in the contralesional hemisphere. Well-recovered patients commonly displayed enhanced activity in the ipsilesional regions and re-establishment of interhemispheric and intra-hemispheric connectivity within the affected hemisphere (Tang et al., 2015; Zheng et al., 2016; Ismail et al., 2024). As a result, many therapies aim to promote these patterns of recovery.

The mechanism of acupuncture and MIT may contribute to bilateral brain activation. Acupuncture stimulates peripheral nerves in a more systemic manner, rather than targeting specific regions like the ipsilesional motor area. His broader stimulation regulates neurotransmitters, promotes neurogenesis and cell proliferation, and controls cerebral blood flow (Cheng, 2014; Chavez et al., 2017). MIT, on the other hand, engages bilateral motor regions during imagery tasks, especially when using the non-dominant hand (Batula et al., 2017). This mental simulation of action activates not only the motor cortex but also broader regions, including the contralesional hemisphere (Mulder, 2007; Batula et al., 2017).

Ipsilesional lateralization after VR therapy may occur because it focuses on rehabilitation through targeted neuroplasticity. VR therapy assigns tasks that require specific motor or sensory function, typically focusing on the affected limhs, prompting the damaged hemisphere (ipsilesional) to “relearn” the tasks repeatedly (Hao et al., 2022).

Music therapy stands out among the four interventions, as it primarily enhances activity in the left hemisphere, regardless of the lesion side. The left temporal regions are known to be involved in auditory processing (Patterson et al., 2002), language (de Heer et al., 2017), and memory functions (Paulesu et al., 1993). Interestingly, a systematic review studying the effect of melodic intonation therapy on non-fluent aphasia stroke patients found more activation in the right hemisphere (Zhang et al., 2021) than in the left hemisphere. This suggests that different methods of delivering the music treatment to the patients would give different results.

### 4.3 Specific regions activated by the adjunctive therapy

The therapies administered to patients are commonly designed to stimulate the affected regions and enhance recovery outcomes. The significance of sensorimotor integration and cognitive processes for recovery after a stroke has also been highlighted in various reviews (Bolognini et al., 2016; Lee and Kim, 2023).

Acupuncture maximizes the utilization of both hemispheres for recovery by engaging ipsilesional motor areas, such as the PreCG, PoCG, SMA, and Cerb, to facilitate motor skill relearning and movement rehabilitation. It also promotes sensorimotor integration through the SFG and SPG involvement, enhancing both functional motor control and cognitive aspects. Additionally, acupuncture utilizes contralesional compensatory mechanism involving the PMd, PreCG, and IPL, further supporting adaptive strategies for movement control during recovery. By engaging both hemispheres, the therapy promotes functional connectivity and coordination, which are associated with better recovery outcomes. The engagement with may be linked to the well-documented itch sensation experienced after acupuncture treatment (Papoiu et al., 2012).

In contrast to acupuncture, MIT highly activates the frontoparietal regions in patients post-treatment. Research indicates that damage to these areas can impair motor imagery abilities, while well-developed frontoparietal connections enhance controllability in motor imagery tasks (Lam et al., 2018; Furuta et al., 2024). This activation may serve as an adaptive strategy to support motor recovery, particularly in subjects with damage to the dominant hemisphere’s corticospinal tract (CST) (Olafson et al., 2022).

Like acupuncture and MIT, VR also engages motor areas and frontal regions, indicating that recovery after a stroke is closely related to the integration of motor function, sensory perception, and cognitive processes. The consistent activation of the PreCG and PoCG across all treatments underscores their importance in stroke rehabilitation. As these therapies focus on specific tasks, the regions activated become increasingly targeted. Interestingly, one study noted that VR treatment activated limbic and subcortical regions associated with emotional regulation and motor control circuits, suggesting that VR may have strong emotional, motivational, or motor coordination components (Hao et al., 2022; Wu et al., 2022).

While music therapy differs by emphasizing the aphasia and language aspects, it still activates the PreCG and PoCG. Recovery from aphasia necessitates involvement of auditory processing and language regions, such as the left STG and MTG, which are clearly engaged by music therapy. This form of therapy particularly enhances the language and default mode networks. Increased clustering following music therapy indicates a promotion of the motor, cognitive, and language systems, stimulating damaged language function areas, regulating neuroplastic changes within the language network, and facilitating the recovery of speech functions (Altenmuller and Schlaug, 2015; Xu et al., 2022).

PreCG and PoCG are consistently reported across various studies as regions where changes in activity levels are correlated with improvements in clinical outcomes. Their importance in stroke recovery stems from their roles as key regions involved in motor and sensory processing, both of which are frequently impaired following a stroke. Additionally, many stroke rehabilitation therapies target these regions directly or indirectly, leveraging their capacity for neuroplastic reorganization, which is central to the recovery process.

### 4.4 fMRI as a key measure post-rehabilitation

With the advancement of MRI acquisition and data analysis methods, rs-fMRI showed its potential as a promising tool to study motor and sensory outcome in stroke patients and to evaluate the effects of different interventions. However, its practical application in clinical settings is still quite limited. The high cost and lack of accessibility of fMRI restrict the number of studies that can be conducted across diverse populations or long-term therapies, ultimately affecting the generalizability of findings and hindering follow-up research. Additionally, some patients, particularly those with motor disabilities, may struggle to remain still for extended periods, which is necessary for obtaining quality fMRI data. This limitation further narrows the scope of fMRI use for specific patient populations and therapies. Moreover, the absence of standardization in both implementation and analysis presents additional challenges for using fMRI in clinical practice, as discussed in the review by Specht (2019).

### 4.5 Limitation

It is important to acknowledge the limitations of this review. Firstly, it should be noted that several articles included in this review originated from the same research group or utilized the same dataset, potentially introducing a degree of bias and reducing the generalizability of the findings. Secondly, the limited number of articles and sample sizes hindered the ability to conduct robust meta-analyses, which could have provided more comprehensive insights into the effectiveness of the interventions. Thirdly, the inclusion criteria restricted the review to articles published in English within the past 10 years, potentially leading to selection bias and excluding relevant studies published in other languages or outside the specified time frame. Besides that, although both control and treatment groups receive “conventional” therapy, the specifics of what constitutes conventional rehabilitation varied between studies. This makes it challenging to determine if the observed effects are due solely to the complementary therapy or an interaction between therapies. However, as our review only includes RCTs, this provides a robust framework to compare complementary therapies while controlling for these confounding factors.

## 5 Conclusion

In conclusion, this systematic review explored the brain activating abilities and patterns of acupuncture, MIT, VR, and music in stroke patients. While acupuncture and MIT promoted bilateral activation, VR therapy favored ipsilesional lateralization, and music therapy focused on left-lateralization. The review highlighted the treatment abilities in enhancing motor, cognitive and sensory areas for achieving better recovery outcomes.

## Data Availability

The original contributions presented in the study are included in the article/supplementary material further inquiries can be directed to the corresponding author.
